# Activities and sources of income after a period of long-term sick leave - a population-based prospective cohort study

**DOI:** 10.1186/1471-2458-12-745

**Published:** 2012-09-06

**Authors:** Anders Wikman, Michael Wiberg, Staffan Marklund, Kristina Alexanderson

**Affiliations:** 1 Division of Insurance Medicine, Department of Clinical Neuroscience, Karolinska Institutet, Stockholm, SE-171 77, Sweden

## Abstract

**Background:**

There is limited knowledge about what happens to people after long-term sick leave. The aim of this report was to conduct a prospective study of individuals who were on prolonged sick leave during a particular year, considering their activities and sources of income during subsequent years. To enable comparison of different time periods, we used three cohorts of individuals with different starting years.

**Methods:**

Using data from national registers, three separate cohorts were constructed that included all people living in Sweden who were 20-64 years of age (>5 million) in the years 1995, 2000 and 2005, respectively. The individual members of the cohorts were classified into the following groups based on their main source of income and activity in 1995-2008: on long-term sick leave, employed, old-age pensioner, long-term unemployed, disability pensioner, on parental leave, social assistance recipient, student allowance recipient, deceased, or emigrated.

**Results:**

Most individuals on long-term (> 6 months) sick leave in 1995 were not employed 13 years later. Only 11% of the women and 13% of the men were primarily in employment after 13 years. Instead, a wide range of alternatives existed, for example, many had been granted disability pension, and about 10% of the women and 17% of the men had died during the follow-up period. A larger proportion of those with long-term sick leave were back in employment when 2005 was the starting year for the follow-up.

**Conclusions:**

The low future employment rates for people on long-term sick leave may seem surprising. There are several possible explanations for the finding: The disorders these people may have, might have entailed longstanding difficulties on the labor market. Besides, long-term absence from work, no matter what its causes were, might have worsen the chances of further employment. The economic cycles may also have been of importance. The improving labor market during later years seems to have improved the chances for employment among those earlier on long-term sick leave.

## Background

Cross-sectional data constitute the main source of official statistics on sickness absence [1,2]. These data are often compiled on an annual basis. Time trends derived from such data provide only rather crude information about changes in sickness absence over time. For example, it can be difficult to determine, whether an increase in sick-leave days in a population is due to longer periods of absence in the same number of people or to a larger number of people with the same length of absence. More generally, cross-sectional data cannot provide information about what happens over the years to individuals who once have been sickness absent [3].

On the other hand, by using micro data in epidemiological cohort studies, individuals can be followed over long periods of time. However, thus far, such studies have mainly included selected and often small groups, and most have been performed to study future sick-leave periods among people with specific health problems, types of rehabilitation, or attitudes [4-14]. A few broader cohort studies have been carried out, such as the 10-Town study in Finland [15], the KIRUT study in Norway [16], the Whitehall II study in the United Kingdom [17], the DREAM study in Denmark [18], the GAZEL cohort study in France [19], and the Östergötland study in Sweden [20]. However, these studies have included only some parts of the total population in their respective countries (e.g. selected geographical areas or occupations). The results of such analyses can rarely be generalized to the general population.

An additional restriction in most longitudinal studies is that the focus is limited to single outcomes. Examples could be continued long-term sickness absence, disability pension, or employment. Such restrictions causes problems when attempting to interpret reported changes, as the alternatives are many. When a sick-leave spell has ended, the individual might have returned to work or perhaps instead become unemployed, begun to study, received disability or old-age pension, or died. It is also possible that the individual has become entirely dependent on someone else’s income. Thus, a more precise understanding of people’s situations after a period of long-term sick leave, requires the inclusion of a wide range of outcomes.

Most previous studies have found large gender differences in the risks of sickness absence [2]. Hence, it is of particular interest to analyze women and men separately in this context.

The aim of our study was to prospectively follow women and men in Sweden who had been on long-term (>6 months) sick leave in 1995, considering their main type of income source and activities during each year of the period 1996–2008. Moreover, the aim was to compare the results when 1995 was taken as the starting year with the results when 2000 and 2005, were the starting years. The importance of age and level of education were also taken as a focus in the analysis.

## Methods

Three cohorts were used in this study: all individuals aged 20–64 years and registered as living in Sweden on 31 December in 1994 (N = 5 092 434) constituted the 1995 cohort and the corresponding populations in 1999 (N = 5 173 076 ) and 2004 (N = 5 277 713 ) constituted the 2000 and 2005 cohorts. The individuals in each of the cohorts were followed prospectively up to 2008 by use of annual data obtained from the registry LISA compiled by Statistics Sweden and from the Death Register. LISA contains information on annual income from gainful employment, on various social security sources, and on unemployed searching for work. Thus, the 1995 cohort could be followed for 13 years, the 2000 cohort for 8 years, and the 2005 cohort for 3 years.

For each year studied, the individuals were classified into different categories based on their main activity or source of income that year. The starting point for this categorization was the way that Statistics Sweden had classified people as being employed or not employed, based on the size of their annual income from work. If a person’s income from work exceeded some minimal amount, he or she had been categorized (according to that classification) as employed. Statistics Sweden has chosen minimum income amounts (which varies between groups) to render data consistent with other statistics [21].

The data were supplemented with information on alternative activities and sources of income, such as the number of days of receiving different types of social security compensation (e.g., sickness or parental leave benefit or disability pension) and the number or days registered as unemployed (adding days when a person actively search for a job according to the Swedish Public Employment Service or was in labor market training or in employment program). If a person had one of these particular income sources or activities for more than six months (full or part time) during a specific year, this was considered the individual’s primary income/activity that year. It was not possible to obtain information about the number of days that individuals had received means-tested social assistance, student allowance, or old-age pension. Therefore, those who were not employed (according to the definition used by Statistic Sweden) were categorized as having received means-tested social assistance, if more than half of their disposable income during a given year consisted of compensation of that type. The same method was applied to individuals with student allowance or old-age pension. Two additional groups were also included in the study: those who died and those who emigrated during the follow-up period.

The classification was made in mutually exclusive groups, which means that each person could be assigned to only one category each year. The advantage in classifying people into mutually exclusive groups is that it makes it possible to follow changes in individuals’ activities and sources of income from one year to another.

Table 1 presents the number and proportion of women and men in each of the categories in 1995. The names given to the categories in this table are used throughout the article.

**Table 1:** Main activities or sources of income among women and men living in Sweden in 1995

		**Women**		**men**	
	**Number**	**%**	**Number**	**%**	
Employed	1 540 076	61.4	1 740 752	67.4
Old-age pensioners	31 227	1.2	33 307	1.3
Long-term unemployed	252 341	10.1	332 982	12.9
Disability pensioners	207 172	8.3	167 416	6.5
Persons on long-term sick leave	71 004	2.8	54 401	2.1
Persons on long-term parental leave	116 811	4.7	3 758	0.1
Social assistance recipients	31 236	1.2	34 702	1.3
Student allowance recipients	83 570	3.3	69 409	2.7
Unemployed without welfare assistance	174 428	7.0	147 842	5.7
All	2 507 865	100	2 584 569	100

20–64 years of age.

The last category in Table 1 (designated “unemployed without welfare assistance”) could be seen as a residual category including individuals, who was not assigned to any of the other categories. Their situation could be said to be unclear; it was only known that they did not have any substantial income from gainful employment or support from welfare assistance.

Individuals’ mobility between categories over time was strongly dependent on age. Most of the old-age pensioners were ≥65 years old, because 65 is the normal age for such retirement in Sweden. Disability pension was more common in those who were in their early sixties whereas parental leave benefit was more common among young women. To illustrate the importance of age we together with other findings present results for one specific age group: the women and men who were 50 years old in 1995.

Considering future activities and income sources among those on long-term sick leave, it is plausible that level of education also played a role, since that factor might influence the opportunities to obtain and keep jobs [22]. To address that issue, logistic regression analyses were used to calculate odds ratios (ORs) for future employment among those with and without long-term sick leave, controlling for age and level of education. Individuals who had had no long-term sick leave during the year of inclusion were in that analysis used as a reference group. The regression analyses were restricted to people aged 20–60 years, divided into eight five-year categories. Level of education was considered in five groups: primary/lower secondary (≤9 years), upper secondary, post secondary (<2 years and ≥2 years), and postgraduate. All analyses were stratified by sex.

Conditions on the labor market and rules regarding eligibility for different welfare benefits has varied over time. The multiple cohort design enabled analysis of cohort effects, i.e. the impact of using different starting years (1995, 2000, and 2005, respectively) and following different time periods. Our data allowed us to compare three different three-year periods in these three different cohorts.

The study received ethical approval from the Regional Ethical Review Board in Stockholm (Dnr: 2007/762-31).

## Results

### Activities and sources of income in 1996–2008 among people on long-term sick leave in 1995

**Figure 1:**
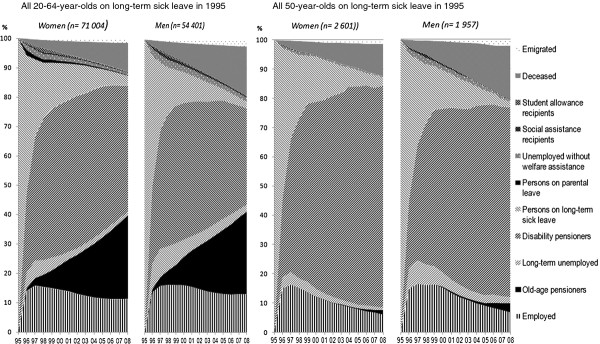
**Activities and sources of income in 1995–2008 among those who were long-term sickness absent in Sweden in 1995.** By sex. 20–64 and 50 years old, respectively. The x-axis represents the years studied (1995–2008).

Over 70 000 women and 50 000 men in Sweden were on long-term sick leave (>6 months) in 1995 (Table 1 and Figure 1). Among those individuals, only 11% of the women and 13% of the men were mainly in employment 13 years later. Many left the labor market and were granted disability pension; this applied to 42% of the women and 40% of the men after two years, and 52% of the women and 47% of the men after five years.

Compared to men, a larger proportion of women was later on long-term sick leave also in the following years. After two years, this was the case for 26% of the women and 23% of the men. Many people went on old-age pension in the follow-up period: 28% of both women and men after 13 years. Few of those with long-term sick leave in 1995 later received long-term unemployment benefits: only 6% of the women and 10% of the men after two years. During the follow-up period, 10% of the women and 17% of the men died.

Among those who were aged 50 years and on long-term sick leave in 1995, only 12% of the women and 15% of the men were employed five years later, and corresponding figures were even lower in the years after that (6% among women and 7% among men after 13 years). Disability pension became relatively predominant in this group and increased with time (76% of the women and 65% of the men after 13 years). The risk of premature death during the 13-year follow up was also comparable high; 11% of the women and 18% of the men died during the follow-up period. This was much higher than for the entire population of the same age (5% of women and 8% of men).

### Comparisons of the three cohorts with starting years 1995, 2000, and 2005, respectively

In general in Sweden, the years covered by the study were characterized by improvement of labor market conditions with time, noted as higher employment and lower unemployment rates [23]. There were also dramatic increases in long-term sick leave during the years between 1997 and 2004 [2], which were accompanied by a number of amendments of the social security legislation [24]. Also some changes in demographic structure was seen through increasing numbers of older persons and immigrants. To ascertain whether these changes had an effect on subsequent activities and income sources among people on long-term sick leave, the 1995 cohort was compared with the corresponding cohorts starting in 2000 and 2005, respectively. Inasmuch as data were available only up to 2008, the follow-up time was limited to three years in these comparisons. For each of the three cohorts, the number of people in each category during the initial three years of follow up are given in Table 2.

**Table 2:** Activities and sources of income among those long-term sickness absent 1995, 2000, and 2005

	**Starting 1995 (women n = 71 004; men, n = 54 401)**			**Starting 2000 (women, n = 129 161; men, n = 77 621)**			**Starting 2005 (women, n = 126 718; men, n = 72 518)**		
	**1996**	**1997**	**1998**	**2001**	**2002**	**2003**	**2006**	**2007**	**2008**
**Women**									
Employed	14.0	15.8	15.3	11.4	14.2	14.9	17.7	24.4	27.8
Old-age pensioners	0.9	2.5	4.2	0.8	2.4	4.4	1.0	3.0	5.5
Long-term unemployed	5.3	6.1	4.9	1.9	2.4	2.4	3.3	3.6	3.6
Disability pensioners	27.3	42.3	48.2	22.3	41.1	50.0	23.2	37.0	41.2
Persons on long-term sick leave	46.7	26.1	19.0	59.4	35.0	22.9	50.4	26.4	15.4
Persons on parental leave	1.9	1.3	1.1	1.4	1.1	1.0	1.6	1.5	1.4
Unemployed without welfare assistance	1.8	2.1	1.9	1.2	1.4	1.4	1.4	1.7	1.9
Social assistance recipients	0.3	0.5	0.5	0.1	0.1	0.2	0.1	0.2	0.3
Student allowance recipients	0.4	0.8	1.3	0.5	0.6	0.4	0.4	0.6	0.6
Deceased	1.3	2.2	2.9	0.9	1.6	2.2	0.8	1.4	1.9
Emigrated	0.2	0.3	0.5	0.1	0.2	0.3	0.1	0.2	0.3
**Men**									
Employed	13.8	16.0	16.3	11.7	14.0	14.6	17.5	23.8	26.2
Old-age pensioners	1.1	3.0	4.9	1.0	3.2	5.8	1.5	4.3	7.5
Long-term unemployed	8.9	9.7	8.3	3.7	4.8	4.8	6.3	6.6	6.4
Disability pensioners	26.3	40.2	45.2	23.4	41.3	48.7	22.8	36.1	39.1
Persons on long-term sick leave	44.5	23.3	15.7	56.2	31.0	18.9	47.9	23.3	13.0
Persons on parental leave	0.1	0.0	0.0	0.1	0.1	0.1	0.1	0.1	0.1
Unemployed without welfare assistance	2.3	2.6	2.6	1.8	1.7	2.0	1.8	2.1	2.5
Social assistance recipients	0.7	1.1	1.1	0.3	0.5	0.6	0.3	0.5	0.7
Student allowance recipients	0.3	0.5	0.9	0.3	0.4	0.3	0.2	0.2	0.3
Deceased	1.9	3.1	4.2	1.5	2.7	3.8	1.5	2.6	3.6
Emigrated	0.2	0.5	0.7	0.1	0.3	0.4	0.1	0.3	0.5

By sex. 20-64 years old. Three different cohorts used with three different starting year.

Due to the large number of participants, all differences exceeding a few tenths of a percent are significant on the 95% level; in cases with very low percentages, differences less than one tenth of a percent are significant.

The initial number of people on long-term sick leave was higher in the 2000 and 2005 cohorts than in the 1995 cohort. For example, 129 161 women had such absence in the 2000 cohort compared to 71 004 in the 1995 cohort. The proportion of people who were on long-term sick leave also the years after the starting year, decreased in all three cohorts, slightly more among men than women. The proportion of people with such absence after three years was lowest in the 2005 cohort. At the same time, employment was higher in this last cohort. As a comparison, 15.3% of the women and 16.3% of the men in the 1995 cohort were classified as employed after three years, and the corresponding figures for the 2005 cohort were 27.8% and 26.2% (i.e. about 10 percent units higher).

A slightly larger proportion of women than of men was granted disability pension after three years in all three cohorts. The proportion on disability pension was not as high in the 2005 cohort as in the other two cohorts.

### Associations between long-term sick leave and later employment, adjusted for age and level of education among women and men

Table 3 presents calculated odds ratios for employment in 1998, 2003, and 2008, respectively, among all women and men included in the three cohorts (with starting years 1995, 2000, and 2005, respectively). The logistic model used in the calculation utilizes the occurrence of long-term sick leave during the starting year as independent variable, together with age and level of education. People with no sickness absence or with shorter absence (<6 months) were used as reference, as were those 31–35 years of age (with many in the beginning of their labor market career) and those with only primary/lower secondary education. The statistical model implied simultaneous control for all variables included in the table.

**Table 3:** Employment rates and odds ratios after three years in three cohorts starting with 1995, 2000 and 2005

	**Cohort 1995, n**	**% employed in 1998**	**OR (95% CI) 1998**	**Cohort 2000, n**	**% employed in 2003**	**OR (95% CI) 2003**	**Cohort 2005, n**	**% employed in 2008**	**OR (95% CI) 2008**
***Women***									
On long-term sick leave (>6 months)									
No (Ref.)	2 255 328	66.67	1	2 231 897	68	1	2 244 034	71.67	1
Yes	65 735	16.38	0.09 (0.09–0.09)	117 959	16.04	0.08 (0.08–0.08)	113 721	30.13	0.16 (0.16–0.16)
Age in starting year									
20–25 years	334 059	56.77	0.65 (0.64–0.65)	297 638	59.9	0.66 (0.65–0.66)	298 795	63.29	0.66 (0.65–0.66)
26–30 years	303 881	61.92	0.78 (0.77–0.78)	286 907	65.76	0.80 (0.79–0.81)	261 182	69.93	0.80 (0.79–0.81)
31–35 years (Ref.)	291 819	67.16	1	307 864	68.96	1	296 330	73.34	1
36–40 years	283 380	73.38	1.40 (1.38–1.42)	294 525	72.16	1.24 (1.23–1.25)	314 533	76.41	1.28 (1.27–1.30)
41–45 years	288 178	75.21	1.60 (1.58–1.62)	284 498	73.19	1.36 (1.34–1.37)	298 470	76.13	1.32 (1.30–1.33)
46–50 years	323 912	73.56	1.56 (1.54–1.58)	287 583	70.3	1.21 (1.20–1.23)	286 031	73.46	1.17 (1.15–1.18)
51–55 years	275 504	66.57	1.19 (1.18–1.20)	320 446	64.07	0.97 (0.95–0.98)	286 330	67.79	0.9 0(0.89–0.92)
56–60 years	220 330	42.82	0.46 (0.45–0.46)	270 395	47.81	0.51 (0.50–0.51)	316 084	53.88	0.51 (0.51–0.52)
Level of education									
Primary lower secondary (≤ 9 years) (Ref.)	504 920	50.10	1	388 871	48.01	1	311 051	47.22	1
Upper secondary	1 176 542	65.55	1.92 (1.90–1.93)	1 185 375	64.92	1.93 (1.91–1.94)	1 139 270	68.22	2.32 (2.30–2.34)
Post-secondary (<2 years)	120 559	67.53	2.32 (2.29–2.36)	128 214	65.18	1.92 (1.90–1.95)	143 078	69.86	2.46 (2.43–2.50)
Post-secondary (≥ 2 years)	512 064	78.77	3.36 (3.33–3.39)	637 284	76.74	3.39 (3.36–3.42)	749 478	79.44	4.07 (4.04–4.11)
Postgraduate	6 978	78.06	3.04 (2.87–3.23)	10 112	77.5	3.22 (3.07–3.38)	14 878	79.16	3.58 (3.43–3.73)
***Men***									
On long-term sick leave (> 6 months)									
No (Ref.)	2 350 276	73.01	1	2 360 584	74.4	1	2 366 019	78.21	1
Yes	49 396	17.63	0.08 (0.08–0.08)	68 253	16.17	0.07 (0.07–0.07)	62 382	29.14	0.12 (0.12–0.12)
Age in starting year									
20–25 years	347 672	66.89	0.61 (0.60–0.62)	310 571	67.7	0.51 (0.50–0.51)	312 779	72.53	0.51 (0.50–0.51)
26–30 years	318 854	76.52	0.98 (0.97–0.99)	297 184	78.93	0.88 (0.87–0.90)	269 869	81.82	0.83 (0.82–0.84)
31–35 years (Ref.)	305 771	76.39	1	322 640	80.2	1	305 433	84.12	1
36–40 years	294 014	77.37	1.09 (1.07–1.10)	307 637	78.32	0.93 (0.92–0.94)	327 724	83.51	1 .00(0.98–1.01)
41–45 years	297 273	77.27	1.11 (1.10–1.13)	293 834	77.41	0.91 (0.90–0.92)	309 668	80.61	0.86 (0.85–0.87)
46-50 years	331 770	76.59	1.11 (1.10–1.13)	294 610	75.13	0.83 (0.82–0.84)	293 398	78.21	0.77 (0.76–0.78)
51–55 years	285 155	70.65	0.85 (0.84–0.86)	325 752	70.22	0.67 (0.66–0.67)	291 146	73.23	0.6 0(0.59–0.61)
56–60 years	219 163	46.31	0.31 (0.30–0.31)	276 609	52.5	0.31 (0.31–0.32)	318 384	49.49	0.32 (0.32–0.33)
Level of education									
Primary/Lower secondary (≤ 9 years) (Ref.)	605 325	62.04	1	497 738	61.5	1	422 340	62.99	1
Upper secondary	1 197 023	72.24	1.5 0(1.49–1.51)	1 249 456	73.4	1.55 (1.54–1.56)	1 248 122	77.56	1.83 (1.82–1.85)
Post-secondary (< 2 years)	202 211	79.06	2.05 (2.02–2.07)	193 367	76.95	1.69 (1.67–1.71)	197 471	79.99	2 .00(1.97–2.03)
Post-secondary (≥ 2 years)	372 795	81.31	2.35 (2.32–2.37)	462 719	80.83	2.31 (2.29–2.33)	531 896	83.53	2.57 (2.55–2.60)
Postgraduate	22 318	84.07	3.04 (2.93–3.15)	25 557	83.23	2.84 (2.75–2.94)	28 572	82.55	2.42 (2.34–2.50)

By sex. All person in Sweden 20-60 years old. Consideration taken to age groups, levels of education, and having or not being on long-term sick leave during the starting year.

Women who had long-term sick leave in 1995 were at 11 times lower risk of being employed in 1998 compared to other women (OR = 0.09). The corresponding OR for the 2000 cohort was the same. For the 2005 cohort, the ratio was slightly higher (OR = 0.16), indicating a reduced difference between the women who had had and those who had not had long-term sick leave during the starting year. The same pattern was found for men.

It seems that age had a greater impact on future employment in the first (1995) cohort compared to the other two cohorts. However, the opposite appeared to be the case regarding level of education, which tended to be more important in the two later cohorts, especially for women. The odds ratios for employment increased with age up to the middle age and did so more pronounced in the 1995 year cohort than in the later cohorts. The odds ratios for employment increased with education especially in later years and especially for women (with the post graduated men as a visible exception.)

## Discussion

We conducted a population-based prospective cohort study by analyzing annual data on future activities and sources of income for people ones long-term sickness absent. The design of the study made it possible to follow the outcomes for each individual in detail. The result pointed at further long-term sick leave and disability pensioning for many. Together, these two categories constituted the dominating outcome, although a broad array of other outcomes also must be taken into consideration, including the following: some were granted old-age pension; some could be classified as unemployed; some chose to further their education; some were on parental leave; many had died. Notably, only a small portion of those who were on long-term sick leave in 1995 could be classified as having employment in subsequent years. Although there were many changes in people’s income sources/activities, they seldom led directly or indirectly to employment. This suggests that long-term sick leave is a very potent risk factor for future worklessness in both the short and the long term.

Three different cohorts with different starting years were compared: 1995 with 2000 and with 2005. The three cohorts gave essentially the same results, with the exception that the risk of future worklessness was lower in the 2005 cohort.

The fact, that most people on long-term sick leave did not return to work in the follow-up period can be interpreted in different ways. Long-term absence from work, no matter what the causes were, might have worsen the chances of further employment [25,26]. It is also possible that the disorders that this people have had, might have entailed longstanding difficulties on the labor market.

It seems that partial support for the latter interpretation is provided by our results concerning negative health outcomes among those who had had long-term sick leave. Many of them were granted disability pension on the basis of medical assessment showing permanent work incapacity. Furthermore, compared to the population in general, a comparatively larger proportion of individuals who had been on long-term sick leave died during the follow-up period. Some people with long-term sick leave apparently had recurring or chronic health problems.

The percent returning to work may be seen low compared to some results from clinical studies of participants in rehabilitation programs [27-29]. It should be noted, however, that our data gives the general statistical picture over several years, behind which could be hidden some flows both into and out of the labor market. People may return to work for a period and then leave again. It should also be noted that those with long-term sick leave were comparable old with a mean of 47 years of age at the first study year. In the last study year they were 13 years older. With time their health problems may have increased.

At the same time it is frequently reported that an association exists between lower educational level and impaired health [22]. Lower educational qualifications can be seen as a risk factor for future impaired health. It also seems reasonable to assume that lower educational qualifications reduce the spectrum of possible occupational alternatives, which will in turn lower the chances of later employment. Educational level could be seen as an underlying cause behind some of our results.

The results of the study indicate that the impact of educational merits on the possibility of procuring employment became increasingly important during later years. There are several possible explanations for this observation. In general, the unemployment levels in Sweden have been much higher during the last decades compared to earlier years, which has generated greater competition for existing jobs. The economic crisis at the beginning of the 1990s was a divide with at first a rapid increase in the levels of unemployment and a then remaining high levels during the years that followed. The last two decades have at the same time been characterized by substantial mobility and rapid transformations among enterprises, which has led to frequent layoffs. Many people who lost their jobs have had problems finding new ones. It seems that this continuous industrial restructuring has raised the demand for skilled labor, and thus made it even more difficult for individuals with low educational qualifications to find suitable employment [30].

The number of appropriate jobs varies over time in relation to the economic cycles, in the sense that more people return to work or get new jobs when times are good and many jobs are available. This may also be valid for those on long-term sick leave. Our comparisons showed higher levels of return to work in the 2005 cohort than in the 1995 or 2000 cohorts, which might have been due to the improved economic situation in Sweden in 2006–2008. The early 2000s did not produce more new employment among the long-term sickness absent than the late 1990s. In Sweden, an improvement in the labor market during the end of the 1990s was followed by some new labor market problems during the dotcom crises in the beginning of the new millennium, but after that the situation on the labor market improved.

The positive return-to-work figures after three years in the 2005 cohort, compared to the 1995 and 2000 cohorts, might also be explained by administrative changes that were made. In 2003, the Swedish government had formulated a goal of achieving a 50% reduction in the number of sick-leave days. The years before, at the end of the 1990s and beginning of the 2000s, there had been a large increase in sick-leave days and this was considered to be partly due to delays in assessments of eligibility for disability pension, which resulted in many people being on sick leave for several years. A few years later, there was instead a comparatively large number of individuals who were granted disability pension; in 2004, the number awarded such compensation reached a maximum at nearly 75 000, which is twice as many as during the years immediately preceding 2000 [31]. Consequently, some cases involving sickness absence caused by more severe impairments were included in the 2000 cohort but not in the 2005 cohort. This gave slightly different 2005 cohort consisting of individuals who on the average were more likely to find work. The administrative efforts (during the 2000s) to reduce the number of sick-leave days may also in it selves have had an influence on the number of return to work.

Notwithstanding, the association between a period of long-term sick leave and subsequent worklessness was strong in all the three cohorts and over all the years we studied, even after adjusting for age and level of education.

### Methodological aspects

A major strength of this study is that it is based on very large cohorts, each comprising more than five million people, including the entire population of working ages in Sweden in 1995, 2000, and 2005, respectively. Those on long-term sick leave in the starting year in each of these cohorts were followed during the subsequent years up to 2008. According to our knowledge, the study is the largest one to have considered outcomes in terms of income sources/activities in people who have been on long-term sick leave. Furthermore, compared to earlier studies, we used a more multidimensional approach to investigate people’s situations after long-term sick leave. Additional strengths are that we had access to annual follow-up data for each cohort member for up to 13 years. All individuals could be followed without any dropouts, and the cohorts were large enough to allow separate analyses of women and men. The fact that three different cohorts could be compared made it possible to examine some crucial cohort effects.

A limitation of our study is that people were categorized as having had only one main activity or source of income during each year. This entailed an approximation; for example, those who were on sick leave for three months during one year were categorized without consideration to that type of information. In future studies, more detailed information about different activities and sources of income need to be considered.

## Conclusions

The low future employment rates for people on long-term sick leave may seem surprising. There are several possible explanations for the finding: The disorders these people may have, might have entailed longstanding difficulties on the labor market. Besides, long-term absence from work, no matter what the causes were, might have worsen the chances of further employment The economic cycles may also have been of importance. The improving labor market during later years seems to have improved the chances for employment among those earlier on long-term sick leave.

## Competing interests

The authors declare that they have no competing interests.

## Authors’ contribution

The study design and major analyses was done by AW. MW assisted in the analyses. AW wrote the first and consecutive drafts of the manuscript. All authors contributed to the interpretation and in writing the manuscript. All authors read and approved the final manuscript.

## Pre-publication history

The pre-publication history for this paper can be accessed here:

http://www.biomedcentral.com/1471-2458/12/745/prepub
